# DEVELOPMENT OF A MOBILE INTERVENTION TO SUPPORT HEALTHY EATING IN PERSON WITH CO-OCCURRING FRAILTY AND DEMENTIA

**DOI:** 10.1093/geroni/igac059.2217

**Published:** 2022-12-20

**Authors:** Oleg Zaslavsky, Kuan-Ching Wu, Shao-Yun Chien, Kimiko Domoto-Reilly

**Affiliations:** University of Washington, Seattle, Washington, United States; University of Washington, Seattle, Washington, United States; University of Washington, Seattle, Washington, United States; University of Washington, Seattle, Washington, United States

## Abstract

New behavioral solutions are needed to improve health in persons with co-occurring frailty and dementia. Using dementia-specific principles of human-centered design, we developed a mobile intervention that includes a patient-facing app and clinician interface to promote a Mediterranean-style eating plan in this population. Our design processes were as follows. We first solicited iterative input from experts in dementia, accessible design, and technology concerning language, cognitive load, and overall accessibility needs in persons with early dementia. Next, we recruited seven people with mild dementia and their partners for interviews, experience sampling, and usability studies. Our primary findings were: 1) participants have basic knowledge of healthy eating tenets and want to learn more; 2) participants are unaware of anything specific to improve in their diet; 3) participants value simple meals with few ingredients; 4) participants strongly rely on physical cookbooks. Based on the results, we developed a high-fidelity prototype of the patient-facing mobile app, divided into tasks: 1) First time onboarding; 2) Setting the first dietary goal; 3) Finding a recipe; 4) Filter/sort for recipes; 5) Food tracking tool; 6) Mastering a dietary goal and starting a new one; 7) Reviewing progress. The resultant product was iterated in usability studies and informed a final prototype for further testing. In parallel, a web-based clinician interface was developed through individual interviews, a survey of potential end-users (n=24), and two rounds of usability studies (n=3 in each round). The online clinician product features these interactive modules: dashboard, tracking manager, patient portal, and individual patient page

